# Proteomic and Ultrastructural Analysis of Cellulite—New Findings on an Old Topic

**DOI:** 10.3390/ijms21062077

**Published:** 2020-03-18

**Authors:** Giamaica Conti, Nicola Zingaretti, Domenico Amuso, Elena Dai Prè, Jessica Brandi, Daniela Cecconi, Marcello Manfredi, Emilio Marengo, Federico Boschi, Michele Riccio, Roberto Amore, Eugenio Luigi Iorio, Alice Busato, Francesco De Francesco, Valentina Riccio, Pier Camillo Parodi, Luca Vaienti, Andrea Sbarbati

**Affiliations:** 1 Department of Neurosciences, Biomedicine and Movement Sciences, Anatomy and Histology division, University of Verona, 37134 Verona, Italy; 2 Accademia del Lipofilling, Research and Training Center in Regenerative Surgery, 61025 Montelabbate, Italy; zingarettin@gmail.com (N.Z.);; 3 Clinic of Plastic and Reconstructive Surgery, Academic Hospital of Udine, Department of Medical Area (DAME), University of Udine, 33100 Udine, Italy; 4 Master of Aesthetic Regenerative and Anti-Aging Medicine, University of Verona, 37134 Verona, Italy; 5 Department of Biochemistry, University of Verona, 37134 Verona, Italy; 6 Department of Sciences and Technological Innovation, University of Eastern Piedmont, 15121 Alessandria, Italy; 7 Department of Informatics, University of Verona, 37134 Verona, Italy; 8 Department of Reconstructive and Hand Surgery, AOU Ospedali Riuniti, 60020 Ancona, Italy; 9 Veternirary Medical School, University of Camerino, 62024 Camerino, Italy

**Keywords:** adult stem cells, mesenchymal stem cell, cellulite, proteomic analysis, dermal adipose tissue

## Abstract

Background: Cellulite is a condition in which the skin has a dimpled lumpy appearance. The main causes of cellulite development, studied until now, comprehends modified sensitivity to estrogens, the damage of microvasculature present among dermis and hypodermis. The differences of adipose tissue architecture between male and female might make female more susceptible to cellulite. Adipose tissue is seen to be deeply modified during cellulite development. Our study tried to understand the overall features within and surrounding cellulite to apply the best therapeutic approach. Methods: Samples of gluteal femoral area were collected from cadavers and women who had undergone surgical treatment to remove orange peel characteristics on the skin. Samples from cadavers were employed for an accurate study of cellulite using magnetic resonance imaging at 7 Tesla and for light microscopy. Specimens from patients were employed for the proteomic analysis, which was performed using high resolution mass spectroscopy (MS). Stromal vascular fraction (SVF) was obtained from the samples, which was studied using MS and flow cytometry. Results: light and electron microscopy of the cellulite affected area showed a morphology completely different from the other usual adipose depots. In cellulite affected tissues, sweat glands associated with adipocytes were found. In particular, there were vesicles in the extracellular matrix, indicating a crosstalk between the two different components. Proteomic analysis showed that adipose tissue affected by cellulite is characterized by high degree of oxidative stress and by remodeling phenomena. Conclusions: The novel aspects of this study are the peculiar morphology of adipose tissue affected by cellulite, which could influence the surgical procedures finalized to the reduction of dimpling, based on the collagen fibers cutting. The second novel aspect is the role played by the mesenchymal stem cells isolated from stromal vascular fraction of adipose tissue affected by cellulite.

## 1. Introduction

Cellulite is the most common modification of skin appearance in women, causing them to seek aesthetic treatments [1]. The scientific literature about cellulite consists of a large number of papers, in which different hypotheses about the origin of all skin modifications were formulated [2,3,4]. Despite the numerous efforts of researchers and aesthetic surgeons, the initial phenomenon that leads to the cascade of events causing the orange peel characteristic and the modification of the subcutaneous compartment, is still matter of debate in the scientific community [5]. There are three principal theories about cellulite’s etiology. The first theory, formulated by Nürnberger and Muller, is based on the different architectural structure of subcutaneous tissues in male and female [6,7]. Using TC, they described the female subcutaneous and dermal compartment as constituted by collagen fibers organized in rectangular lobules surrounded by branches of collagen disposed perpendicularly to the skin surface, while male subcutaneous and dermal compartments are characterized by the deposition of collagen fibers randomly disposed in the tissue, in order to form small polygonal lobules [7]. Nürnberger and Muller confirmed, in females, the thickening of collagen fibers that surround the lobules, affecting its protrusion, which is composed by mature adipocytes compressed inside each lobule. This mechanism is influenced by estrogen causing the protrusion of these *papillae adiposae* visible only in women [2,7].

The second theory, formulated by Merlen and Curri, is based on the hypothesis of vascular changes. The authors described a different pattern of lymphatic drainage and blood circulation in cellulite-affected tissue that leads to the development of fibrosis [8,9]. The third theory, formulated by Gruber and Huber and Draelos attributes the development of cellulite to the chronic inflammation subsequent to the estrogens’ action and to the deposition of glycosaminoglycans (GAGs) by dermal fibroblasts [10,11].

Our paper aims to improve the knowledge about cellulite insurgence and development of studying the morphology of adipose tissue affected by cellulite; the novel aspects could influence the surgical procedures finalized to the reduction of the affected area. We studied cellulite affected tissues with a multimodal approach: magnetic resonance imaging (MRI), ultrastructural analysis (Transmission Electron Microscopy (TEM) and Scanning Electron Microscopy (SEM)) and proteomics both of cellulite tissue and MUSE (multi-lineage differentiating stress enduring) cells, a subpopulation of mesenchymal stem cells that are stress-tolerant and pluripotent, with special regenerative capability [12,13,14]. We identified these cells in cellulite-affected tissue and these findings pave the way for further studies aimed to investigate how these stem cell subpopulations play a role in the cellulite etiology. These cells are characterized by a high regenerative capability and could have a role in the dermis adipose tissue modification during the early phases of cellulite development. In fact, we observed MUSE cells closed to mature unilocular adipocytes and to sweat glands. Their quantity in cellulite affected tissue suggest a pivotal role of MUSE cells in this pathology. To our knowledge, this is the first study of cellulite proteome and it allowed to characterize the first step of the cascade of events implicated in cellulite development. In the present study, the authors investigated samples of tissue affected by cellulite excised from cadaver and biopsies of women subjected to surgical treatments to remove orange peel characteristics on the skin. The samples of tissue excised from cadavers were examined by magnetic resonance imaging to verify the structure of subcutaneous and dermal compartment, while biopsies collected from patients were employed for the isolation of mesenchymal stem cells and for proteome analysis.

## 2. Results

### 2.1. MRI of Cellulite

**Figure 1:**
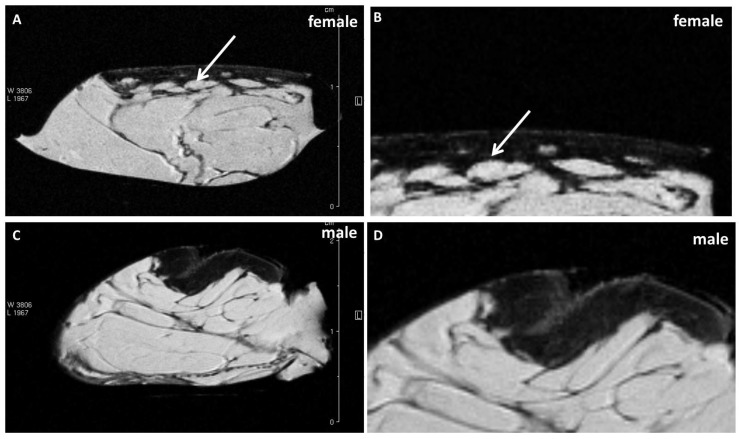
Cellulite MRI. (**A**,**B**): Cellulite of female patients. (**C**,**D**): Cellulite of male patients. The figure shows the organization of collagen fibers in the dermis of female and male. It is possible to observe a non-uniform distribution of collagen bundles both in female and in male. The main difference is represented by the dimension of adipose lobules surrounded by collagen fibers, larger in men and better defined by collagen septa. Moreover, the presence of dermal papillae was detectable only in the female dermis and not in male, indicating a different organization that could be related with cellulite development (**A**,**B**).

**Figure 2:**
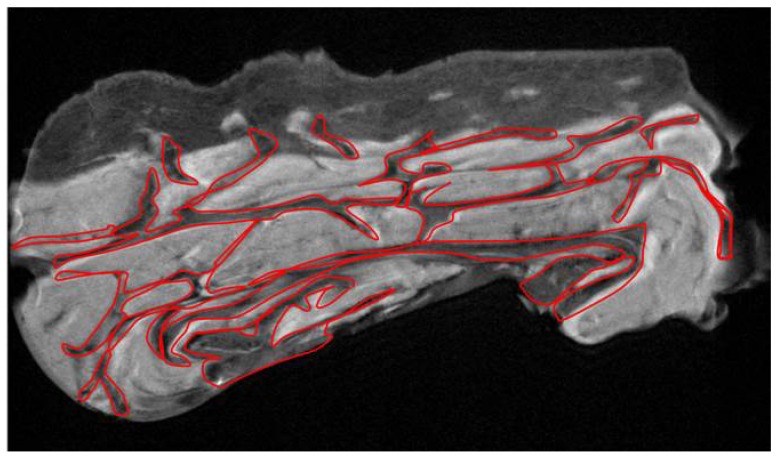
Quantification of collagen fibers: on MRI slices, it was possible to determine the area occupied by collagen fibers. Specifically, we manually tracked a region of interest corresponding to the collagen fibers. We examined 10 slices for each patient and calculated the mean value for each patient, the mean value between all the patients, and the standard deviation. Examining 10 different slices allowed us to determine the amount of collagen in the whole specimen. Moreover, it was possible to determine the number of visible collagen septa in 10 slices for each sample.

MRI analysis revealed different features about the macroscopic aspect of cellulite affected tissues. The subcutaneous and dermic architecture was similar between female and male, with non-uniform distribution of collagen fibers within the compartments and surrounding fat lobules (Figure 1). In the female acquisition, the adipose lobules in subcutaneous tissue appeared better organized and of homogeneous dimensions; in fact, the collagen fibers formed a mesh characterized by well-structured limited adipose tissue lobules (Figure 1A,B). In men, the disposition of collagen fibers appeared more randomly organized and the adipose lobules assumed different forms and dimensions (Figure 1C,D). Furthermore, in men, the collagen bundles are detectable in the dermis, while the female dermis presented small depots characterized by the same signal intensity of adipose tissue (Figure 1A,B white arrows). On MRI slices, the quantification of the area occupied by collagen fibers was performed (Figure 2). The Table 1 showed the mean value of collagen fibers number for each patient, derived from 10 different quantifications of fibers visible in the samples harvested from n = 5 men and n = 5 women. Moreover, the area occupied by collagen fibers was quantified in 10 different field of view for each patient and the mean values were reported in the Table 1. Figure 2 shows the region of interest (ROI) manually tracked in order to identify collagen fibers.

### 2.2. SEM and TEM of Cellulite

**Figure 3:**
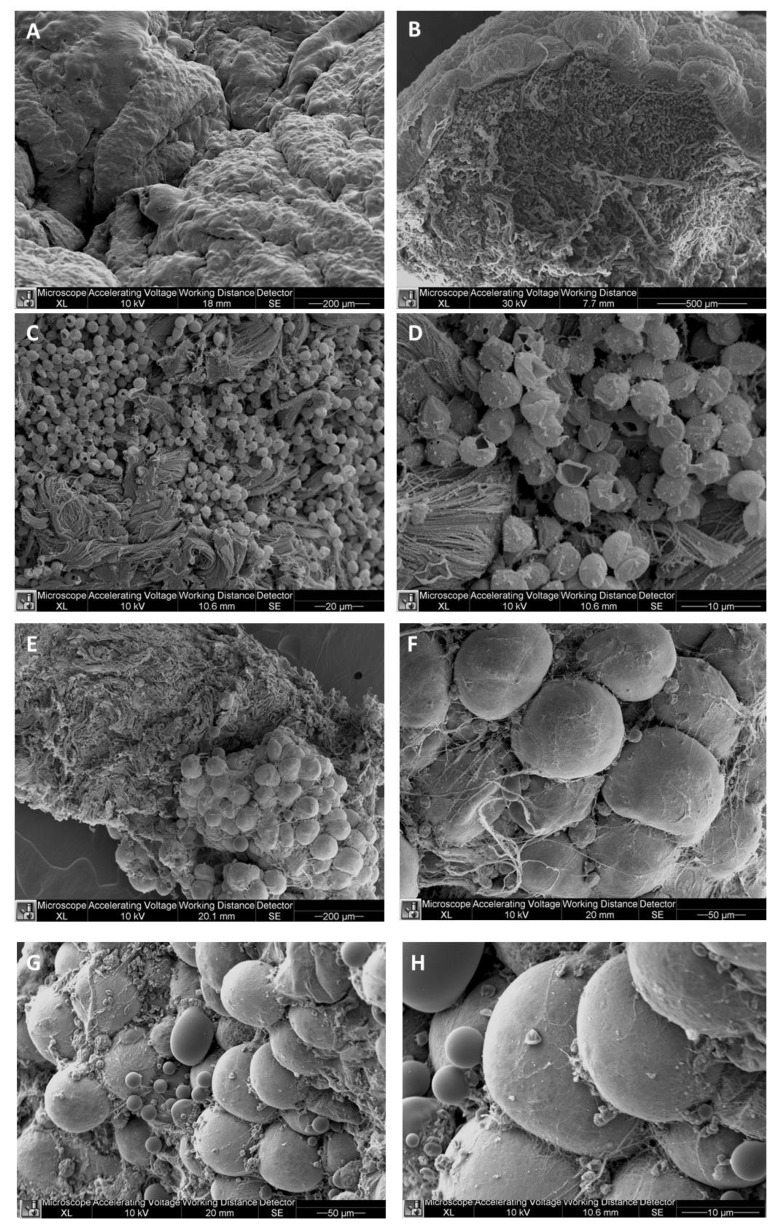
SEM of cellulite. With SEM, it is possible to observe cellulite specimens from the skin to the hypodermis. In (**A**,**B**), it is possible to appreciate the skin with an “orange peel” characteristic, while in the deeper layers, the adipocytes clusters are visible and are surrounded by thick collagen bundles (**C**,**D**). In **D**, it was possible to observe the abundance of collagen around the lobules of adipocytes, which at higher magnification appeared to be covered by very thin but dense collagen scaffold (**E**,**F**). (**G**,**H**): SEM of the gluteal femoral area of man in which the different organization of collagen matrix and bundles are visible. In men, the lobules are less regular in dimension and shape. They are surrounded by thinner collagen bundles.

In the present study, cellulite specimens, harvested postmortem from five female and five male patients, were observed from the skin layer to the subcutaneous compartment. Using SEM, it was possible to observe the dimpling formation on the skin with the characteristic features of cellulite affected tissue (Figure 3A,B). In all the specimens it was possible to observe the presence of deflated mature adipocytes, characterized by loss of lipid charge. Moreover, the thick collagen fibers were detectable among the deflated adipocytes in the subcutaneous layer (Figure 3B,C). The dermal layer was characterized by a different organization of the subcutaneous compartment where the adipocytes were organized in limited clusters embedded in a dense matrix of thick collagen boundless (Figure 3D,E). This organization correspond to the small depots detectable in magnetic resonance imaging in the dermo- hypodermic junction, which could be defined as dermis papillae (Figure 3F). Moreover, the SEM performed on samples of skin harvested from men showed a completely different pattern (Figure 3G,H). In fact, it is possible to appreciate the presence of mature adipocytes surrounded by thin collagen fibers instead of being clusterized.

**Figure 4:**
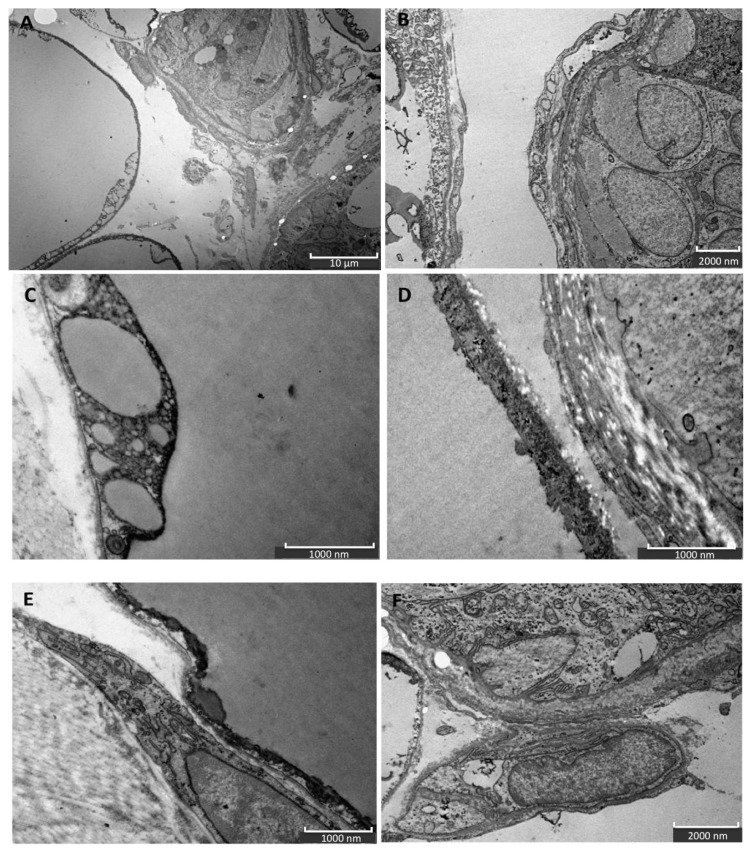
TEM of cellulite. With TEM, it is possible to observe the functional unit of cellulite, composed of sweat glands and mature unilocular adipocytes. In the extracellular space, micro vesicles are detectable (**A**,**B**); they resemble small lipid droplets. Moreover, some vesicles are present in extracellular space, indicating a possible communication between mature adipocytes and sweat glands. The TEM also showed highly activated membrane of mature adipocytes, in which numerous lipid droplets are present (**C**,**D**). Some stem cells are visible near the complex formed by adipocytes and sweat glands, and are recognizable for the big nucleus and for the paucity of organelles in the cytoplasm (**E**,**F**).

With TEM, it is possible to observe the functional unit of cellulite, composed by sweat glands and mature unilocular adipocytes (Figure 4A,B). Moreover, mature adipocytes plasmatic membranes could be activated because of the presence of lipid droplets, which probably represent vesicles secreted in the extracellular space (Figure 4C,D). TEM analysis of the subcutaneous compartment also evidenced the presence of fibroblasts, close to the mature adipocytes, and a high number of stem cells, characterized by a large nucleus and a cytoplasm poor of organelles and mitochondria (Figure 4E,F).

### 2.3. Mesenchymal Stem Cells Isolation

**Figure 5:**
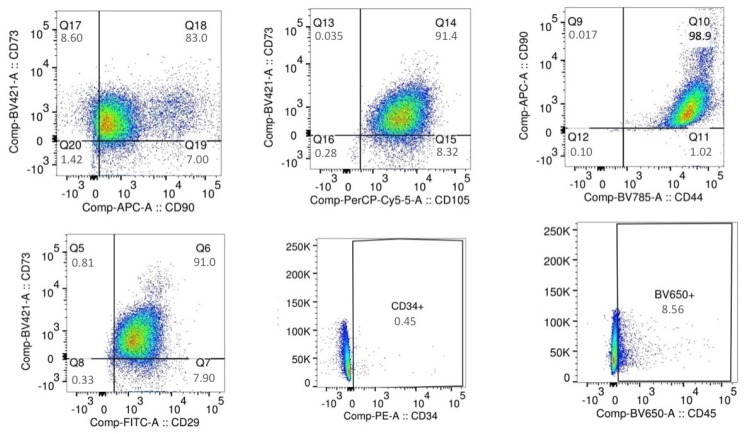
MSCs flow cytometry. Using flow cytometry and the panel usually employed for the characterization of mesenchymal stem cells, we demonstrated that the cells isolated from cellulite specimens are mesenchymal cells and express the stem markers. The upper line shows the dot plots that indicate the positivity for the expression of mesenchymal stem cell markers CD73, CD90, and CD105, compared to each other. The lower line shows the dot plots that indicate the negative expression of hematopoietic markers CD 29, CD34, and CD45, compared with the expression of CD73.

On the basis of the ultrastructural findings, regarding the presence of stem elements, specimens of cellulite were digested and mesenchymal stem cells were isolated. Flow cytometry confirmed the mesenchymal origin by the positive expression of CD73, CD105; CD90, CD44, and CD29, which are typical mesenchymal stem cells markers (Figure 5). Figure 5 shows the negative expression of hematopoietic markers.

### 2.4. MUSE Purification

**Figure 6:**
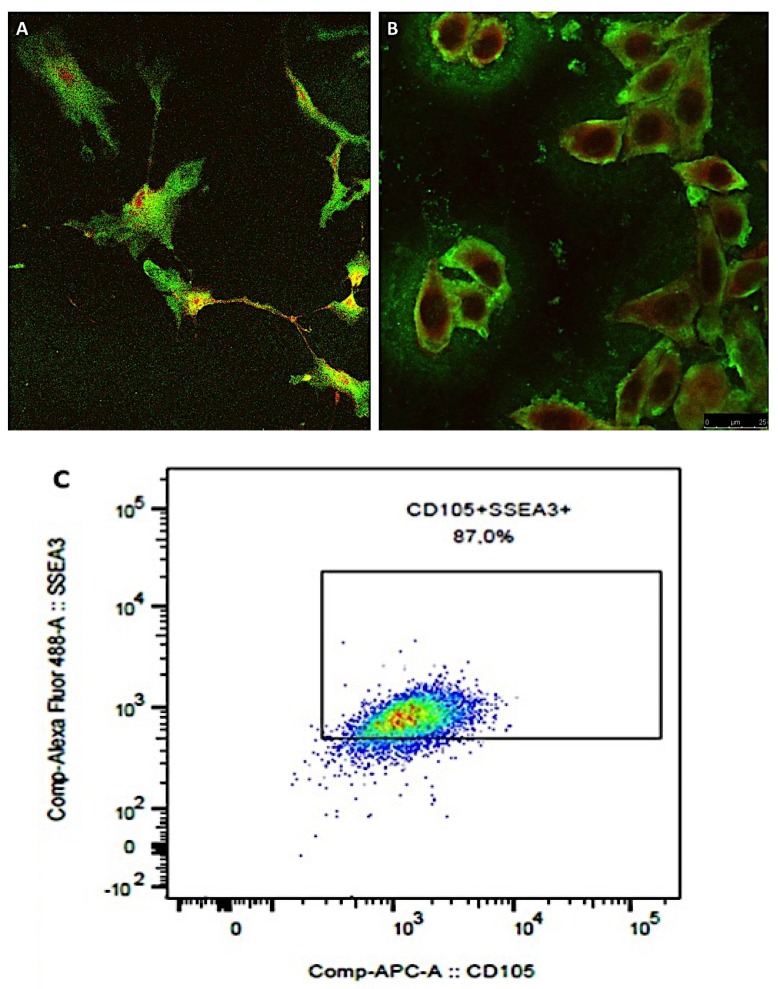
Confocal microscopy of MUSE cells. The presence of MUSE cells has been observed in mesenchymal stem cell culture, incubated with anti-SEEA3 and anti-CD105 antibodies. Numerous cells have been characterized by the simultaneous expression of CD105 (red) and of SEEA3 (green) and could be classified as MUSE cells. (**A**) and (**B**) showed the MUSE cells imaged 72 h after isolation and 7 days of culturing. The percentage of MUSE cells has been determined by flow cytometry, with immune sorting (**C**). MUSE cells were characterized by the simultaneous expression of CD105 and SEEA3 at a high level.

Using two different techniques, it was possible to show the presence of multilineage differentiating stress enduring cells as a sub population of isolated mesenchymal stem cells. Confocal microscopy performed labeling cells with both anti-SEEA3 and anti-CD105 antibodies showed the presence of cells that simultaneously expressed these two markers, indicating the existence of MUSE cells sub population (Figure 6A,B). Flow cytometry cell immune sorting performed labeling of the total population of mesenchymal stem cells isolated from cellulite specimens, with anti-SEEA3 and anti-CD105 antibodies. This confirmed the presence of high amounts of MUSE cells, which represented almost 85–90% of the mesenchymal stem cell population (Figure 6C). The differences of the MUSE cells quantified in different patients were analyzed by an ANOVA test, and the results were statistically significant with a *p*-value of 0.002, indicating the relationship between the MUSE percentage and the level of cellulite.

### 2.5. Proteomic Analysis of Cellulite and MUSE Cells Isolated from Cellulite Affected Tissue

In order to investigate the role played by the MUSE cells subpopulation in cellulite, a proteomic analysis was performed on the tissue affected by cellulite and on the MUSE cells from two biological replicates. A total of 683 proteins were identified in the MUSE cells and 140 proteins in the adipose tissue, with a peptide confidence cut-off of 99% (FDR < 1%); among them, 61 proteins were found to be common to the two samples. These proteins are reported in Supplemental Table S1.

**Figure 7:**
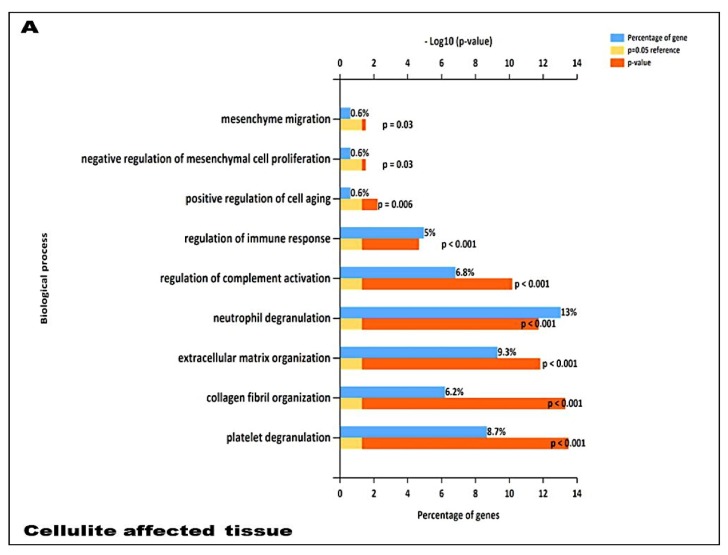

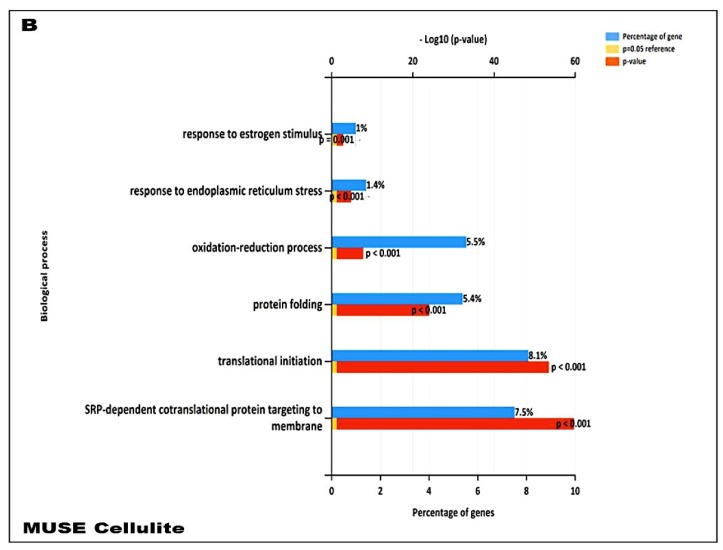
Proteomics of cellulite. (**A**) Proteomics of the adipose tissue. (**B**) Proteomics of MUSE cells. It is possible to visualize the most important pathways present in adipose tissue affected by cellulite and in MUSE cells. The figure shows the pathways characterized by a percentage of genes higher than 1%, which represent the most important families of proteins.

In order to more deeply investigate all the molecular mechanisms involved in cellulite and in the MUSE cells, further bioinformatics analyses were carried out: they provided information on the biological process, the subcellular localization, and the molecular function of all the proteins involved in cellulite development and in all the changes of tissue architecture that leads to the typical “orange peel” characteristic. The most represented biological processes in cellulite affected tissue were: extracellular matrix organization (10.7%), complement activation, (9.3%), innate immune response (9.3%), and platelet degranulation (9.3%) (Figure 7A). Concerning MUSE cells, a high number of proteins were involved in cell-cell adhesion (11.7%), in translation (9.3%), in SRP-dependent co-translational protein targeting to membrane (8%), and oxidation-reduction process (6.8%) (Figure 7B). Moreover, other important families of proteins were detected in cellulite-affected tissue and MUSE cells, but expressed in a lower percentage (less than 1%), such as the response to reactive oxygen species and the response to estrogen or estradiol.

Functional annotation analysis also revealed the presence of proteins mainly at the level of extracellular exosomes, and showed to be implicated in protein binding both in MUSE and in cellulite affected tissues (Table S2). Interestingly, STRING analysis highlighted several enriched pathways from the Reactome database, observed both in cellulite affected tissues and in the derived stem cells, such as immune system, metabolism, neutrophil degranulation, and vesicle-mediated transport (Table S3).

## 3. Discussion

Many surgeons and numerous papers defined cellulite as an aesthetic problem that requires cosmetic treatments in order to obtain a smooth skin [2,4,6]. Most surgeons and researchers affirmed that cellulite represents the most frequent event in post pubertal women. In fact, all the studies concerning cellulite revealed that about 85–90% of women are affected by an “orange peel” skin characteristic [15,16]. During the last 10 years, published papers focused on the different possible modifications of the subcutaneous adipose tissue that could represent the initial events of cellulite [17,18]. They described disorders in blood capillaries organization, different distribution of collagen fibers in the subcutaneous compartment, impairment of the lymphatic drainage, and, because cellulite is typical only of women, they attributed all these phenomena to estrogen activity [3]. The present study, based on a multimodal approach, aimed to describe cellulite starting from the skin appearance and delving into the description of deeper compartments, in order to identify the possible causes leading to the deep modification observed in the dermis.

Examining the cellulite specimens, collected from different women, it was possible to observe the dimpling on the skin and the organization of subcutaneous and dermal compartments. First of all, using magnetic resonance imaging, it was possible to reveal the presence of small adipose papillae, located in the dermis, that are not present in men. MRI acquisitions allow to determine the area occupied by collagen in each patient, tracking a region of interest on 10 different MRI slices for each patient. These values collected from men and women and examined by MRI confirmed the higher deposition of collagen fiber in women subjected to cellulite, if compared with men. It is not possible to identify the type of collagen incremented in the specimens with cellulite. A specific staining is necessary and will need to be the focus of another paper. Men were characterized by different distribution of collagen fibers that occupied a smaller area of the dermis that the area determined in cellulite samples excised from women. This finding confirms the role of fibroblasts involved in the deposition of a higher amount of collagen in adipose tissue affected by cellulite than in men.

The ultrastructural analysis allowed to investigate the cellulite samples at an ultrastructural level, showing the organization of dermal lobules, composed by mature unilocular adipocytes close to the sweat glands. This represent the first findings about cellulite-affected tissue. A similar morphology was described in breast during pubertal phase, in which the sebaceous glands start to produce adipocytes, under estrogenic stimulation. In cellulite, the sweat gland has the same role.

MRI and ultrastructural results could influence the microsurgical methods proposed until now to treat the dimpling [19,20], or therapies based on radiofrequency [21] and on laser employment [22]. Most of these techniques, in fact, are based on the collagen fiber remodeling or cutting, but we are not sure that the remodeling of collagen septa is the most efficient method to treat cellulite. These techniques have been developed based on the previously theories [6,7] about dermis differences between male and female, which are refuted by the data showed by our results (Figure 1).

In addition, our data suggest that during cellulite development the main role is played by mesenchymal stem cells, which are rich in estrogen receptor and are recruited after stress stimuli, represented by oxidative stress, matrix remodeling, reactive oxygen species production, as demonstrated by proteomic analysis (Figure 7). Cellulite appeared characterized by a high amount of mesenchymal stem cells, a feature related to the stimulation of mesenchymal stem cells that could be represented by the observation of numerous vesicles in the extracellular matrix of the dermis that were detected by transmission electron microscopy, in the interspace between adipocytes and sweat glands. Obviously, the electron microscopy alone is not sufficient to study the significance of these vesicles, but the proteomic analysis, using the functional annotation analysis, confirmed the presence of proteins involved in exosome secretion. Exosomes are lipid vesicles secreted by adipocytes, or by adipose mesenchymal stem cells, with the aim to release molecular mediators, in particular in stress conditions [23], and are considered a critical messenger for cell-cell communication. The relevant presence of exosomes supports the existence of a crosstalk between adipocytes and sweat glands, which leads the cellulite progression. This complex could be defined as dermic papilla and it could be considered a peculiar microenvironment in which all the molecular pathways of cellulite were activated. Moreover, the proteomic analysis of cellulite showed the presence of numerous families of proteins related to oxidative stress and extracellularmatrix remodeling and deposition (Figure 7). The alteration of reactive oxygen species level and a massive extracellular matrix deposition is a frequent feature in many chronic inflammatory processes, supporting the theories of Gruber and Huber [10,11]. Additionally, a correlation between oxidative stress and fibrotic processes are known in skin [24], and ROS can play a crucial role in fibrotic degeneration, including growing stimulation of fibroblasts.

**Figure 8:**
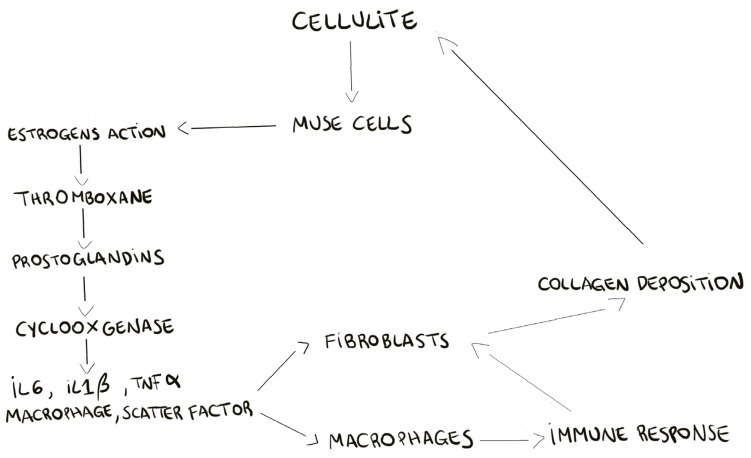
Hypothesis of the events that leads cellulite development. The pivotal role of MUSE cells in cellulite development is summarized in the scheme. In fact, the estrogenic receptors are present only on MUSE cells and not in the adipose tissue. Cellulite development is led by mesenchymal stem cell that are sensitive to estrogens; consequently, the pathway of thromboxane is induced, with a production of COX2, matrix metalloproteinases, and elastase, which induce the strong remodeling of the tissue. Moreover, they stimulate the production of other proteins involved in the immune response.

The proteomic analysis of cellulite and of MUSE cells, isolated from cellulite specimens, showed the presence of estrogens receptors on the multilineage differentiating stress enduring cells membranes, demonstrating the pivotal role of these cells in the cellulite development. According to the previous theories about cellulite progression, our results recognize the importance of estrogenic stimulation [2]. Our hypothesis on MUSE role, is that these cells, after estrogenic stimuli, could activate all the bio chemical cascades of prostaglandins production, cyclooxygenases expression, and of stimulation of matrix metalloproteinases and elastases. A schematic summary is reported in Figure 8, in which we try to clarify the development of cellulite. The recruitment of MUSE, bringing the estrogens receptor, induces the deep remodeling of the dermis and the activation of pro-inflammatory pathways.

Proteomic analysis confirmed numerous aspects that were described in the previous literature about cellulite, such as the presence of proteins involved in tissue remodeling, fibroblast activation, extracellular matrix, and collagen fibers organization. Additional innovative information, provided by the analysis of proteome, is the presence of families of proteins involved in immune system activation in adipose tissue affected by cellulite, which was not observed in the proteome of MUSE cells. Surely, this aspect must be studied with higher attention, in order to comprehend how the immune system could influence cellulite development and progression.

All the collected data provided important findings about cellulite affected tissue, which had never been studied with a multidisciplinary approach, allowed the analysis of phenotypical and molecular aspects. In this study, cellulite was described starting from the skin appearance to the molecular level with the description of pathways involved in this pathological progression.

In previous published studies, in which the cellulite origin were hypothesized, only a single aspect was evaluated: either the vascular aspect, or the organization of collagen fibers, or the formation of adipose tissue papillae. In this paper, the knowledge on cellulite origin was expanded upon, and for the first time, all the aspect of this pathology were analyzed together.

### Limitations of the Study

#### This Study has Some Limitations to Point Out

The dehydration protocol was necessary to obtain slices for morphological and ultrastructural analysis. The dehydration of the specimen may disturb the variation of cellulite in the architecture.

We did not include any images of cellulite-free area in this work. The comparison between cellulite areas and cellulite-free areas was not the goal of this work.

The sample population was small. Larger series are needed to validate these new findings.

## 4. Materials and Methods

### 4.1. Adipose Tissue Harvesting

Cellulite specimens were collected from 10 women (23–45 years old, with a BMI ranging between 24 and 27) that were subjected to surgical treatment, based on the mini invasive protocol previously published by Amore et al. [19]. During the mini invasive surgery, it was possible to harvest punches of 0.5 mm in length and of 0.2 mm of diameter in the site of insertion of the bistoury. Punches were immersed in a 0.9% glucose sterile solution with 0.1% of human insulin and kept at 4 °C.

Others 10 biopsies of gluteo femoral area were surgically collected from cadavers (5 male, 5 female, 25–45 years old, with a BMI ranging between 24 and 27), provided by ICLO Teaching and Research Center, in Verona. Biopsies were employed for the study of cellulite affected adipose tissue phenotype using Magnetic Resonance Imaging, Transmission electron microscopy and Scanning electron microscopy.

### 4.2. Magnetic Resonance Imaging of Cellulite

The specimens of cellulite harvested from cadavers were observed using a spectrometer Bruker Biospin (Bruker Biospin MRI GmBH, Ettlingen, Germany) operating at 7 T, with a strength of 040 G/m and equipped with 3.5 cm i.d. transmitter/receiver birdcage coil. The samples were positioned inside the coil and the region of interest (ROI) was selected in order to collect the whole image of the sample with all the details of epidermidis. The acquisitions were performed using T1-weighted sequences at high resolution with the following parameters: TE = 25 ms, TR = 1168.2 ms, field of view = 3.0 × 3.0 cm, number of averages = 1, flip angle 180 degrees, slice thickness 1.0 mm and matrix size 256 × 256 pixels.

On the slices of MRI, an area corresponding to the space occupied by collagen was manually tracked. The software of the spectrometer provided the millimeters squared, representing collagen fraction of the field of view. Ten field of views were analyzed, in order to determine the mean of the surface occupied by collagen fibers. The analysis of MRI images has been done by two independent expert radiologists, blinded for the gender of the samples.

### 4.3. Ultrastructural Analysis of Cellulite Affected Adipose Tissue

#### 4.3.1. Transmission Electron Microscopy

The samples of cellulite harvested during surgical treatment of cellulite, in living people, were fixed with glutaraldehyde 2% and were post-fixed in 1% osmium tetraoxide (OsO_4_) aqueous solution for 2 h, dehydrated in graded concentrations of acetone and immersed in an Epon–Araldite mixture (Electron Microscopy Sciences, Fort Washington, PA, USA). The semi-thin sections (1 mm thick) were examined by light microscopy and stained with toluidine blue. The ultra-thin sections of 70 nm were obtained using a microtome and placed on Cu/Rh grids with Ultracut E (Reichert, Wien, Austria), stained with lead citrate, and observed using an FEI Morgagni 268 D electron microscope (FEI Company, Eindhoven, Netherlands).

#### 4.3.2. Scanning Electron Microscopy

The specimens of cellulite (10 living female) were fixed with glutaraldehyde 2 % in 0.1 M PB, post-fixed in 1 % osmium tetraoxide (OsO_4_) in the same buffer for 1 h, dehydrated in concentrations of acetone, critical point dried (CPD 030, Balzers, Vaduz, Liechtenstein), fixed to stubs with colloidal silver, sputtered with gold by an MED 010 coater (Balzers, Brugherio, Italy), and examined with an FEI XL30 scanning electron microscope (FEI Company, Eindhoven, Netherlands).

The analysis of SEM/TEM images has been done by two independent experts morphologist, blinded for the gender of the samples.

### 4.4. Purification of Stromal Vascular Fraction from Cellulite

The purification of stromal vascular fraction was performed by digesting fat samples collected from cellulite specimens of 10 living female with collagenase type I 0.2 % in a buffered solution containing 2% of fetal bovine albumin, at 37 °C for 45 min. After digestion samples were transferred in plastic tubes and centrifuged at 400× *g* (3000 rpm) for 7 min. The obtained pellets were suspended in 1 mL of NH_4_CL 160 mM in order to lyse the erythrocytes and then centrifuged at 400× *g* for 10 min. The pellets were suspended in 1 mL of growth medium Dulbecco modified eagle medium (DMEM) added with 10 % of fetal bovine serum (FBS) and 1% of a mixture of penicillin and streptomycin. The number of cells, of each sample, was determined using a Burker chamber, and then cell suspensions were transferred in a flask of 25 cm^2^ with the addition of 5 mL of growth medium. Flasks were incubated at 37 °C and 5% of CO_2_ for 72 h.

### 4.5. Isolation of Mesenchymal Stem Cells and of Multilineage Differentiating Stress Enduring Cells (MUSE) from Cellulite

At the end of the 72 h of initial incubation, with the same medium and in the same conditions described before, cells of each sample were detached using trypsin-EDTA 0.25%, introduced into a plastic tube, and then centrifuged at 400× *g* for 5 min and plated in 75 cm^2^ flasks at 37 °C and 5% of CO_2_, to allow the cells growth. When the cells reached the number of 3 × 10^6^, they were collected and prepared for immune phenotyping and cell sorting, following the procedures described by Conti et al. [25], in order to identify and isolate the mesenchymal stem cells.

For the immune sorting of the mesenchymal stem cells, the following antibodies were employed: APC-conjugated CD90 (dilution 1:5), PerCP-Cyt5.5-conjugated CD105 (dilution 1:20), BV421-conjugated CD73 (dilution 1:20), BV785-conjugated CD44 (dilution 1:20), PE-conjugated CD34 (dilution1:5), FITC-conjugated CD29 (dilution 1:20), and BV650-conjugated CD45 (dilution 1:20). All these antibodies were purchased form BD Bioscences, Becton Dickinson Italy S.p.A., Milan.

At the end of the first phase of isolation, the sorted cells were positive for CD90, CD73, CD105, CD44, and CD29 and negative for CD45 and CD34, being identified as mesenchymal stem cells that were plated on 75 cm^2^ flasks and expanded. When the cells reached the number of 2 × 10^6^, they were detached with tripsin-EDTA 0.25% and collected for the immune sorting for the simultaneous expression of SEEA3 and CD105. The CD105 was used at the same dilution described before, while the Alexa Fluor-488-conjugated SEEA3 antibody was purchased from Aurogene S.R.L (Italy, Rome) and was used at the dilution of 1:200 in volume by distilled water. Immune sorting was performed using a FACS canto II (BD, Becton Dickinson Italy S.p.A Milan).

### 4.6. Confocal Microscopy of MUSE Cells

Confocal microscopy was used to confirm the results of immune sorting where 10^4^ of MUSE cells, isolated from the population of mesenchymal stem cells, were seeded on a glass with a diameter of 24 mm and inserted in six well-plates, and the growth medium previously described was added. The plates were incubated at 37 °C and 5% of CO_2_ for 24 h. At the end of the incubation, the time cells were fixed with 4% buffered formalin for 1 h at 4 °C in dark, and then washed three times with sterile PBS. Successively, the cells were incubated with a PBS solution containing SEEA3 and CD105 antibodies in dark at 4 °C for 30 min. Antibodies were used at the same dilutions described before. At the end of the incubation with the antibodies, MUSE cells were washed with PBS and then added with 50 µL of mounting medium containing DAPI on each slice. The slices were imaged with a Leica SP5 confocal microscope (Leica Microsystems, Mannheim, Germany) with a 40× objective. Images were prepared using Las X software.

### 4.7. Purification of Proteome from Abdomen and Cellulite Affected Adipose Tissue and from Isolated Cells

Adipose tissue sections (10 µm-thick) were cut from the cellulite specimens of 10 living female (20 slices) using a cryostat and placed at −80 °C until use. The samples were placed in 1 mL cold PBS1X added with 1X Complete Mini EDTA-free protease inhibitors (Roche Italia, Monza, Italy) and 1% sodium dodecyl sulphate (SDS) (Biorad, Milan, Italy). The tissue sections were then subjected to 5–6 cycles (each cycle for 30 sec) of sonication. The whole procedure was carried out in cooled conditions. The sample tubes were kept in ice for 30 min and then centrifuged at 14,000× *g* at 4 °C for 15 min. The cell lysate was extracted in the aqueous layer and separated. A fourfold volume of ice-cold acetone was added to the cell lysate, vortexed and incubated at −20 °C overnight. After centrifugation at 14,000× *g* at 4 °C for 10 min, the supernatant was discarded and the pellet was washed with 500 µL of ice-cold acetone-methanol (8:1). For the MUSE cells, the protein extraction was performed in 1 mL of PBS1X, added with protease inhibitors and 1% SDS, through five steps of sonication on ice, each lasting 15 sec. The proteins were subjected to ice-cold acetone precipitation overnight and then recovered after centrifugation at 14,000× *g* at 4 °C for 15 min. The protein pellets were dried and solubilized in 1 mL of 100 mM NH_4_HCO_3_. The protein concentrations were determined by mean of the Bradford reagent (Sigma-Aldrich, Milan, Italy), using bovine serum albumin as standard. Before shotgun proteomic analysis, the proteins were subjected to in-solution digestion as previously described.

### 4.8. Proteomic Analysis

Protein identification by LC-MS/MS analyses were performed on the 10 living female biopsies using a micro-LC Eksigent Technologies (Dublin, USA) system interfaced with a 5600 + TripleTOF system (AB Sciex, Concord, Canada). The stationary phase was a Halo C18 column (0.5 × 100 mm, 2.7 µm; Eksigent Technologies Dublin, USA). The mobile phase was a mixture of 0.1% (v/v) formic acid in water (A) and 0.1% (v/v) formic acid in acetonitrile (B), eluting at a flow-rate of 15.0 µL min with an increasing concentration of solvent B from 2% to 40% in 30 min. Injection volume was 4.0 μL and oven temperature was set to 40 °C. For identification purposes the samples were subjected to data-dependent acquisition (DDA): mass spectrometer analysis was performed using a mass range of 100–1500 Da (TOF scan with an accumulation time of 0.25 s), followed by an MS/MS product ion scan from 200 to 1250 Da (accumulation time of 5.0 ms) with the abundance threshold set at 30 cps (35 candidate ions can be monitored during every cycle). The ion source parameters in electrospray positive mode were set as follows: curtain gas (N2) at 25 psig, nebulizer gas GAS1 at 25 psig, and GAS2 at 20 psig, ion spray voltage floating (ISVF) at 5000 V, source temperature at 450 °C and declustering potential at 25 V [26]. The DDA files were searched using Protein Pilot software v. 4.2 (SCIEX, Concord, Canada) and Mascot v. 2.4 (Matrix Science Inc., Boston, USA). Trypsin as digestion enzyme was specified for both software. For Mascot, we used two missed cleavages, set the instrument to ESI-QUAD-TOF and specified the following modifications for the assay: carbamidomethyl cysteine as fixed modification and oxidized methionine as variable modification. An assay tolerance of 50 ppm was specified for peptide mass tolerance, and 0.1 Da for MS/MS tolerance. The peptide charges to be detected were set to 2+, 3+, and 4+, and the assay was set on monoisotopic mass. The UniProt Swiss-Prot reviewed database containing human proteins (version 2015.07.07, containing 42,131 sequence entries) was used and a target-decoy database search was performed. False Discovery Rate was fixed at 1%.

### 4.9. Bio-Informatics Analysis

The functional annotation analysis of the identified proteins was performed using the Database for Annotation, Visualization and Integrated Discovery (DAVID, v6.8, http://david.abcc.ncifcrf.gov/) [27] (Table S1). The functional annotation chart report was used to identify Gene Ontology (GO) biological processes, molecular function, and cellular component. Gene enrichment analysis was performed by setting the threshold of an EASE Score, a modified Fisher Exact *p*-value, to 0.1, and a *p*-value correction smaller than 0.05 (Table S2).

The identified proteins present in the adipose tissues and in the MUSE cells were analyzed using STRING software (http://string-db.org) [28]. STRING analysis was performed by setting Homo Sapiens as the species under investigation and a high confidence level, namely 0.7; we retrieved interactions based exclusively on experimental and database knowledge, while excluding all other prediction methods implemented in STRING (such as text mining and gene fusion). Reactome pathways were reported by setting FDR < 1% (Table S3).

### 4.10. Human and Animal Rights or Ethical Approval

Written informed consent was obtained from each patient prior to the study. Our institutional ethics committee (Comitato di Approvazione per la Ricerca sull’Uomo—C.A.R.U., University of Verona, Italy) approved the study design (n. 2019-UNVRCLE-0175710).

## 5. Conclusions

The present study could be considered a more complete study about cellulite, because it is the first study about cellulite proteome. The proteomic analysis underlined some innovative aspects not yet examined by researchers. The data collected with proteomic analysis must be studied deeply in order to find the first phenomena that leading to the cellulite development. A multidisciplinary approach has been useful to enrich the knowledge about a very complicated phenomenon involving dermal adipose tissue.

## Figures and Tables

**Table 1:** Quantification of collagen fibers: The mean value among different slices of each patients and the mean value between all the patients were reported in the Table. The difference between men and women is appreciable and correspond to the incremented activity of fibroblast, which has a pivotal role in collagen deposition. SD: standard deviation.

**patient**	**mean value number of fibers**	**mean value of area (mm^2^)**
male 1	37	1542
male 2	42	1658
male 3	38	2140
male 4	41	1874
male 5	47	1652
		
mean	41	1773.2
SD	3.52	212.65
**patient**	**mean value number of fibers**	**mean value of area (mm^2^)**
female 1	67	2584
female 2	58	2386
female 3	49	1988
female 4	54	2106
female 5	64	2685
		
mean	58.4	2349.8
SD	6.52	267.90

## Supplementary Materials

The following are available online at https://www.mdpi.com/1422-0067/21/6/2077/s1.

## References

[1] Sadick N. (2018). Treatment for cellulite. Int. J. Womens Dermatol..

[2] De la Casa Almeida M., Suarez Serrano C., Rebollo Roldán J., Jiménez Rejano J.J. (2013). Cellulite’s aetiology: A review. J. Eur. Acad. Dermatol. Venereol..

[3] Terranova F., Berardesca E., Maibach H. (2006). Cellulite: Nature and aetiopathogenesis. Int. J. Cosmet. Sci..

[4] Pavicic T., Borelli C., Korting H.C. (2006). Cellulite—The greatest skin problem in healthy people? An approach. J. Dtsch. Dermatol. Ges..

[5] Christman M.P., Belkin D., Geronemus R.G., Brauer J.A. (2017). An Anatomical Approach to Evaluating and Treating Cellulite. J. Drugs Dermatol..

[6] Nurnberger F., Muller G. (1978). So-called cellulite: An invented disease. J. Dermatol. Surg. Oncol..

[7] Nurnberger F. (1981). Practically important diseases of the subcutaneous fatty tissue (including so-called cellulite). Med. Welt..

[8] Merlen J.F., Curri S.B. (1984). Anatomico-pathological causes of cellulite. J. MalVasc..

[9] Curri S.B., Merlen J.F. (1986). Microvascular disorders of adipose tissue. J. MalVasc..

[10] Gruber D.M., Huber J.C. (1999). Gender-specific medicine: The new profile of gynecology. Gynecol. Endocrinol..

[11] Draelos Z.D. (2005). The disease of cellulite. J. Cosmet. Dermatol..

[12] Kushida Y., Wakao S., Dezawa M. (2018). Muse Cells Are Endogenous Reparative Stem Cells. Adv. Exp. Med. Biol..

[13] Wakao S., Kushida Y., Dezawa M. (2018). Basic Characteristics of Muse Cells. Adv. Exp. Med. Biol..

[14] Fisch S.C., Gimeno M.L., Phan J.D., Simerman A.A., Dumesic D.A., Perone M.J., Chazenbalk G.D. (2017). Pluripotent nontumorigenic multilineage differentiating stress enduring cells (Muse cells): A seven-year retrospective. Stem Cell Res. Ther..

[15] Khan M.H., Victor F., Rao B., Sadick N.S. (2010). Treatment of cellulite: Part I. Pathophysiology. J. Am. Acad. Dermatol..

[16] Bielfeldt S., Buttgereit P., Brandt M., Springmann G., Wilhelm K.P. (2008). Non-invasive evaluation techniques to quantify the efficacy of cosmetic anti-cellulite products. Skin Res. Technol..

[17] Querleux B., Cornillon C., Jolivet O., Bittoun J. (2002). Anatomy and physiology of subcutaneous adipose tissue by in vivo magnetic resonance imaging and spectroscopy: Relationships with sex and presence of cellulite. Skin Res. Technol..

[18] Gensanne D., Josse G., Theunis J., Lagarde J.M., Vincensini D. (2009). Quantitative magnetic resonance imaging of subcutaneous adipose tissue. Skin Res. Technol..

[19] Amore R., Amuso D., Leonardi V., Sbarbati A., Conti G., Albini M., Leva F., Terranova F., Guida A., Gkritzalas K. (2018). Treatment of Dimpling from Cellulite. Plast. Reconstr. Surg. Glob. Open.

[20] Geronemus R.G., Kilmer S.L., Wall S.H., Green J.B., Cohen J.L., Weiss R.A., Alster T.S., Kaminer M.S., Gupta A. (2019). An Observational Study of the Safety and Efficacy of Tissue Stabilized-Guided Subcision. Dermatol. Surg..

[21] Hexsel D., Fabi S.G., Sattler G., Bartsch R., Butterwick K., Casabona G., Yen-Yu Chao Y., Costa J., Eviatar J., Geister T.L. (2019). Validated Assessment Scales for Cellulite Dimples on the Buttocks and Thighs in Femal Patients. Dermatol. Surg..

[22] Petti C., Stoneburner J., McLaughlin L. (2016). Laser cellulite treatment and laser-assisted lipoplasty of the thighs and buttocks: Combined modalities for single stage contouring of the lower body. Lasers Surg. Med..

[23] Vizoso F.J., Eiro N., Cid S., Schneider J., Perez-Fernandez R. (2017). Mesenchymal Stem Cell Secretome: Toward Cell-Free Therapeutic Strategies in Regenerative Medicine. Int. J. Mol. Sci..

[24] Siems W., Grune T., Voss P., Brenke R. (2005). Anti-fibrosclerotic effects of shock wave therapy in lipedema and cellulite. Biofactors.

[25] Conti G., Bertossi D., Dai Prè E., Cavallini C., Scupoli M.T., Ricciardi G., Parnigotto P., Saban Y., Sbarbati A., Nocini P. (2018). Regenerative potential of the Bichat fat pad determined by the quantification of multilineage differentiating stress enduring cells. Eur. J. Histochem..

[26] Martinotti S., Patrone M., Manfredi M., Gosetti F., Pedrazzi M., Marengo E., Ranzato E. (2016). HMGB1 Osteo-Modulatory Action on Osteosarcoma SaOS-2 Cell Line: An Integrated Study from Biochemical and -Omics Approaches. J. Cell. Biochem..

[27] Huang D.W., Sherman B.T., Lempicki R.A. (2009). Systematic and integrative analysis of large gene lists using DAVID bioinformatics resources. Nat. Protoc..

[28] Szklarczyk D., Gable A.L., Lyon D., Junge A., Wyder S., Huerta-Cepas J., Simonovic M., Doncheva N.T., Morris J.H., Bork P. (2019). STRING v11: Protein-protein association networks with increased coverage, supporting functional discovery in genome-wide experimental datasets. Nucleic Acids Res..

