# Supplementary material for: Proteomic and Ultrastructural Analysis of Cellulite—New Findings on an Old Topic
Source: Int J Mol Sci. 2020 Mar 18;21(6):2077. doi: 10.3390/ijms21062077 (PMC7139738; doi:10.3390/ijms21062077)
Supplement: Supplementary file 1 [file ijms-21-02077-s001.pdf]

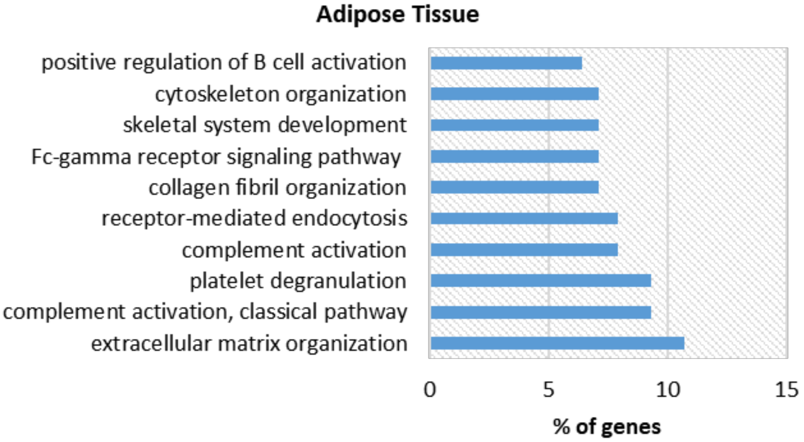

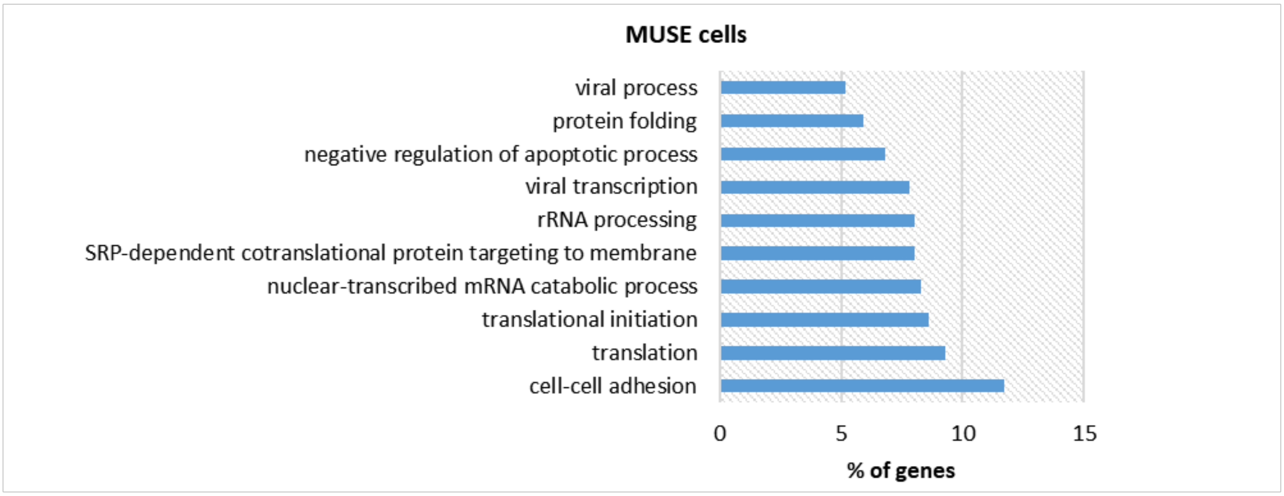

Supplementary S1

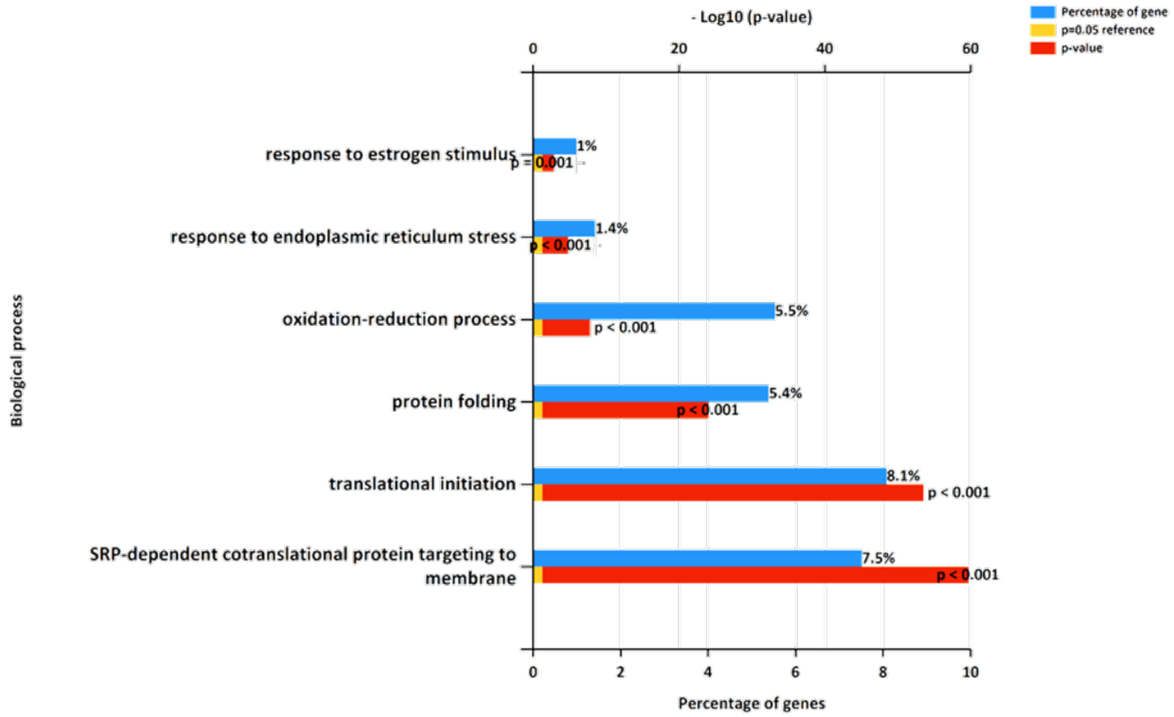

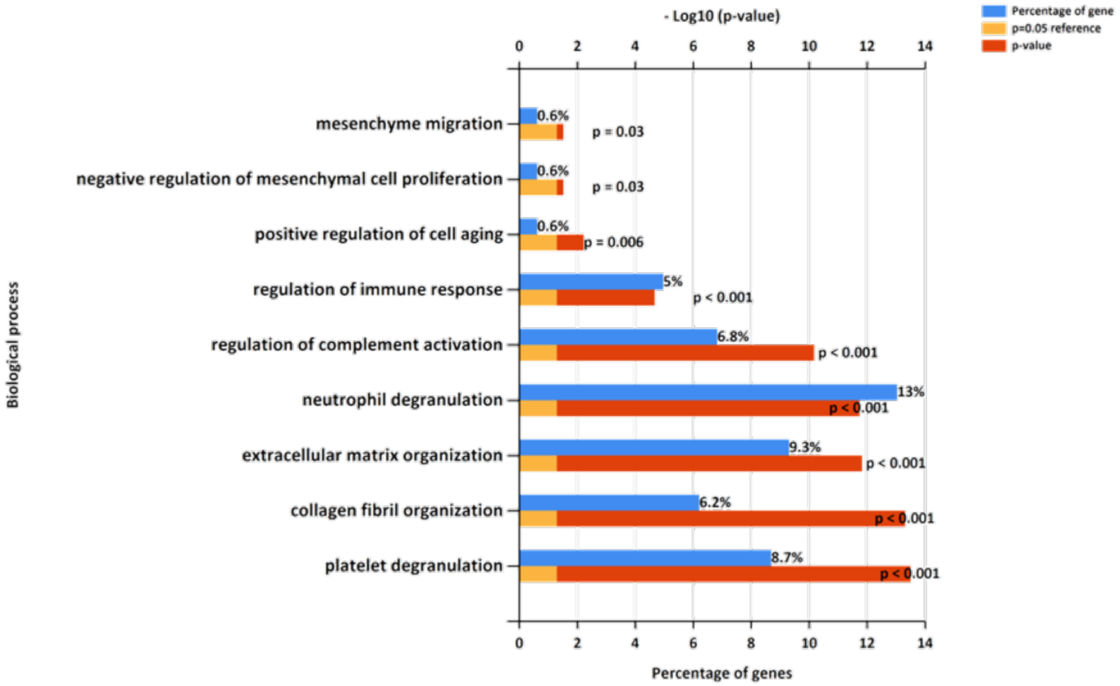

Supplementary S2

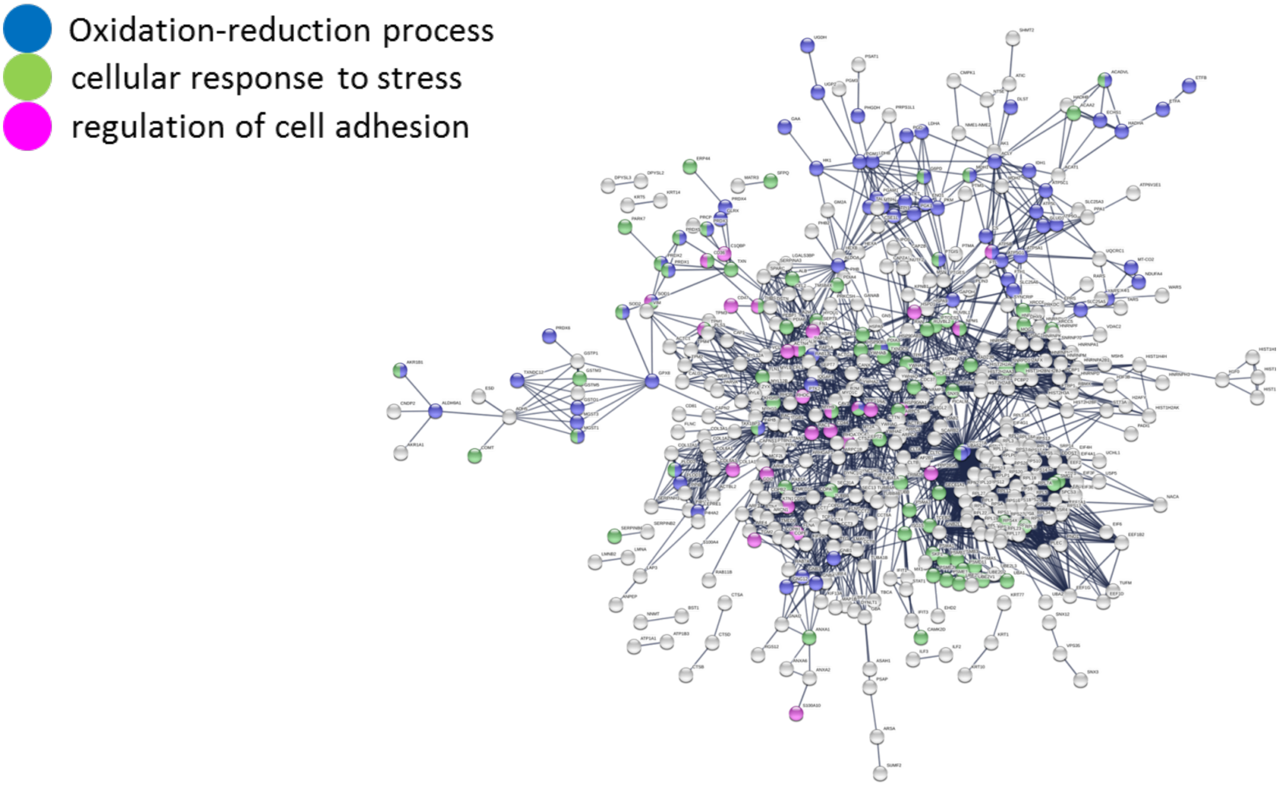

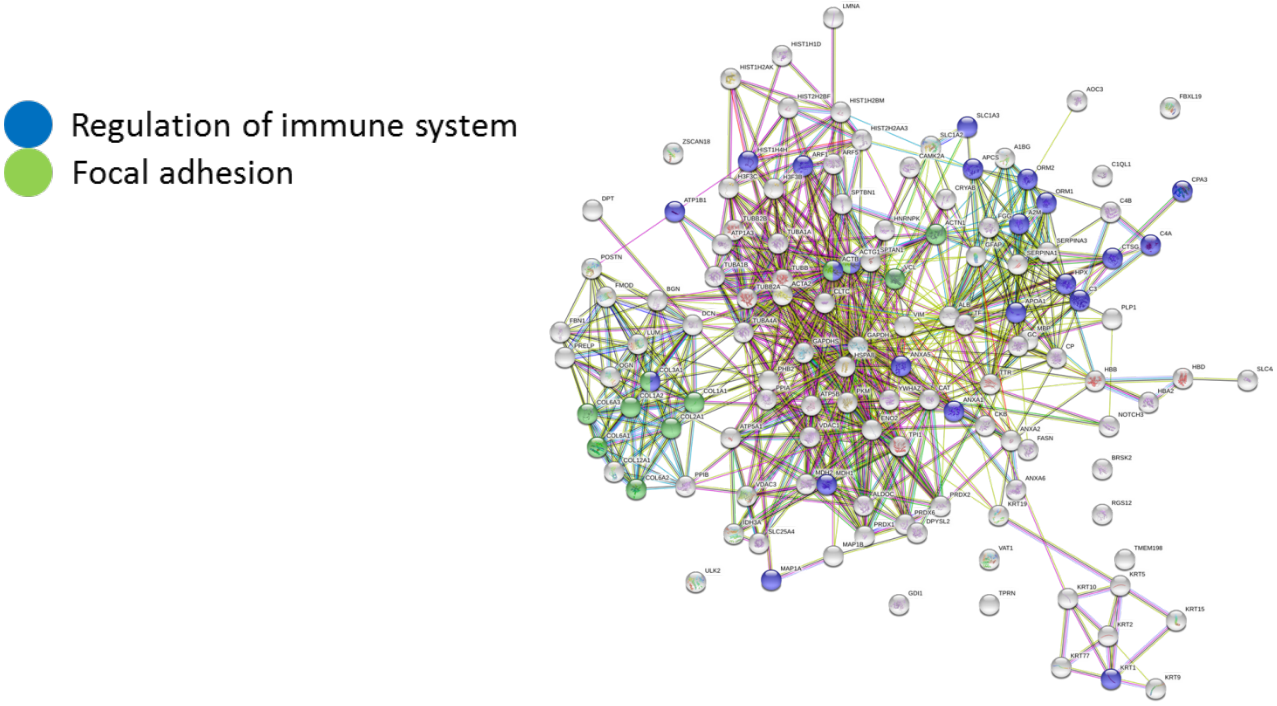

Supplementary S3
